# The presence of rNTPs decreases the speed of mitochondrial DNA replication

**DOI:** 10.1371/journal.pgen.1007315

**Published:** 2018-03-30

**Authors:** Josefin M. E. Forslund, Annika Pfeiffer, Gorazd Stojkovič, Paulina H. Wanrooij, Sjoerd Wanrooij

**Affiliations:** Department of Medical Biochemistry and Biophysics, Umeå University, Umeå, Sweden; Max Planck Institute for Biology of Ageing, GERMANY

## Abstract

Ribonucleotides (rNMPs) are frequently incorporated during replication or repair by DNA polymerases and failure to remove them leads to instability of nuclear DNA (nDNA). Conversely, rNMPs appear to be relatively well-tolerated in mitochondrial DNA (mtDNA), although the mechanisms behind the tolerance remain unclear. We here show that the human mitochondrial DNA polymerase gamma (Pol γ) bypasses single rNMPs with an unprecedentedly high fidelity and efficiency. In addition, Pol γ exhibits a strikingly low frequency of rNMP incorporation, a property, which we find is independent of its exonuclease activity. However, the physiological levels of free rNTPs partially inhibit DNA synthesis by Pol γ and render the polymerase more sensitive to imbalanced dNTP pools. The characteristics of Pol γ reported here could have implications for forms of mtDNA depletion syndrome (MDS) that are associated with imbalanced cellular dNTP pools. Our results show that at the rNTP/dNTP ratios that are expected to prevail in such disease states, Pol γ enters a polymerase/exonuclease idling mode that leads to mtDNA replication stalling. This could ultimately lead to mtDNA depletion and, consequently, to mitochondrial disease phenotypes such as those observed in MDS.

## Author summary

Mitochondria are essential for energy production, and defects in the maintenance of mitochondrial DNA (mtDNA) lead to a variety of human diseases including mtDNA depletion syndrome (MDS). Certain forms of MDS are caused by imbalances in the mitochondrial deoxyribonucleoside triphosphate (dNTP) pool, which have also been shown to lead to altered levels of the ribonucleotides (rNMPs) that are embedded in mtDNA. In this study, we address the impact of these rNMPs on the mitochondrial DNA polymerase Pol γ at nucleotide concentrations that resemble those found inside a cell. We demonstrate that embedded rNMPs do not impair DNA synthesis by Pol γ even at the lowest concentrations of dNTPs tested. Based on these results, an increase in mtDNA rNMPs is unlikely to explain the mitochondrial defects in MDS. However, we find that Pol γ is inhibited by physiological levels of free ribonucleoside triphosphates (rNTPs). When combined with a dNTP pool imbalance, the presence of rNTPs leads to DNA replication stalling by Pol γ. These characteristics of Pol γ may help to explain the mtDNA depletion in forms of MDS.

## Introduction

The replication of DNA is a highly accurate process where free deoxyribonucleoside triphosphates (dNTPs) are incorporated opposite their complementary base. In general, DNA polymerases are good at discriminating between ribonucleoside triphosphates (rNTPs; the units that constitute RNA) and dNTPs by virtue of a steric gate residue that clashes with the 2′-OH group of rNTPs [1]. However, because the rNTP concentration in the cell is several orders of magnitude higher than that of dNTPs, DNA polymerases will occasionally erroneously incorporate rNTPs instead of dNTPs [2]. The presence of embedded ribonucleoside monophosphates (rNMPs) in DNA induces structural and chemical changes [3,4] that contribute to unwanted effects such as genome instability and replication stress [5,6].

Due to their negative influence on DNA stability, rNMPs are actively removed from nuclear DNA (nDNA) by the ribonucleotide excision repair (RER) pathway that is initiated by cleavage at the incorporated rNMP by the enzyme RNase H2 [6,7]. Mutations in RNase H2 lead to an increased rNMP frequency in nDNA and can in humans give rise to rare autoinflammatory disorders [8,9]. However, RER-mediated rNMP removal is absent in mitochondria [10,11]. Accordingly, mammalian mitochondrial DNA (mtDNA) has for decades been known to be rich in rNMPs [12–14]. Recent studies using fibroblast cell lines show that rNMPs are present at a frequency of approximately 54 rNMPs per 16 kb mammalian mtDNA molecule [10]. It is currently unclear whether the rNMPs embedded in mtDNA have a functional significance or if they merely are relatively well-tolerated in the mitochondria and therefore do not undergo the prompt removal observed in nDNA. Nonetheless, mutations in the gene coding for RNase H1, an endonuclease implicated in the removal of longer stretches of rNMPs, cause adult-onset mitochondrial encephalomyopathy marked by multiple mtDNA deletions [15], underscoring the importance of at least a certain level of rNMP removal from mtDNA.

MtDNA is a 16.5 kb circular, double-stranded DNA molecule that encodes for key subunits of the oxidative phosphorylation (OXPHOS) system. OXPHOS is responsible for the majority of the ATP production in eukaryotic cells, and malfunctions in this process can lead to neuromuscular disorders, emphasizing the importance of mtDNA integrity [16]. The duplication of mtDNA is performed by a set of dedicated replication proteins that are nuclear-encoded and post-translationally imported into mitochondria. These include the replicative mtDNA polymerase Pol γ, the replicative helicase Twinkle, mitochondrial single-stranded DNA-binding protein (mtSSB) and the mitochondrial RNA polymerase that primes mtDNA replication [17].

Pol γ discriminates rigorously between dNTPs and rNTPs [18], but the frequency of rNMP incorporation into the genome also depends on the ratio between the free dNTPs and rNTPs available in the cell. The mtDNA is especially vulnerable in this respect, since it is replicated independently of cell cycle phase [19]. Outside S phase, dNTP levels are low, and since rNTP levels show little fluctuation over the cell cycle, the rNTP/dNTP ratio is expected to be high [20,21]. The rNTP/dNTP ratio might be particularly high in certain tissues of patients that suffer from defects in the mitochondrial dNTP supply. Mutations in *e*.*g*. thymidine kinase 2 [22] or deoxyguanosine kinase [23] are expected to lead to decreased mitochondrial pools of certain dNTPs, which in turn leads to mtDNA instability by a still uncertain mechanism. A recent study by Berglund *et al*. suggested that an increased rNTP/dNTP ratio could cause the elevated levels of mtDNA rNMPs observed in fibroblasts derived from patients with defects in mitochondrial dNTP metabolism [10]. It was further proposed that increased rNMP accumulation in mtDNA might impair consecutive rounds of replication and could therefore contribute to the mtDNA instability and disease phenotypes observed in these patients. However, no studies have so far addressed how the fidelity and efficiency of Pol γ are affected by incorporated rNMPs at physiologically relevant dNTP and rNTP levels.

Using highly purified recombinant proteins we show that human Pol γ bypasses single rNMPs with an unprecedentedly high efficiency and fidelity. Additionally, our data indicate that human Pol γ—alone or in the context of the reconstituted mitochondrial replisome—has a striking ability of discriminating against rNMPs during incorporation, and displays several-fold lower frequency of rNMP incorporation compared to nuclear replicative polymerases. However, we show that free rNTPs can negatively impact mtDNA replication, likely by competing with the dNTPs for binding to the active site during DNA synthesis by Pol γ, and can lead to Pol γ stalling. In certain diseases, dNTP pool disturbances lead to compromised mtDNA stability without any major effect on the nDNA. We propose that this differential outcome is at least partly due to the sensitivity of the mitochondrial replication fork to high rNTP/dNTP ratios.

## Results

### Pol γ efficiently replicates DNA templates containing single embedded rNMPs

**Fig 1:**
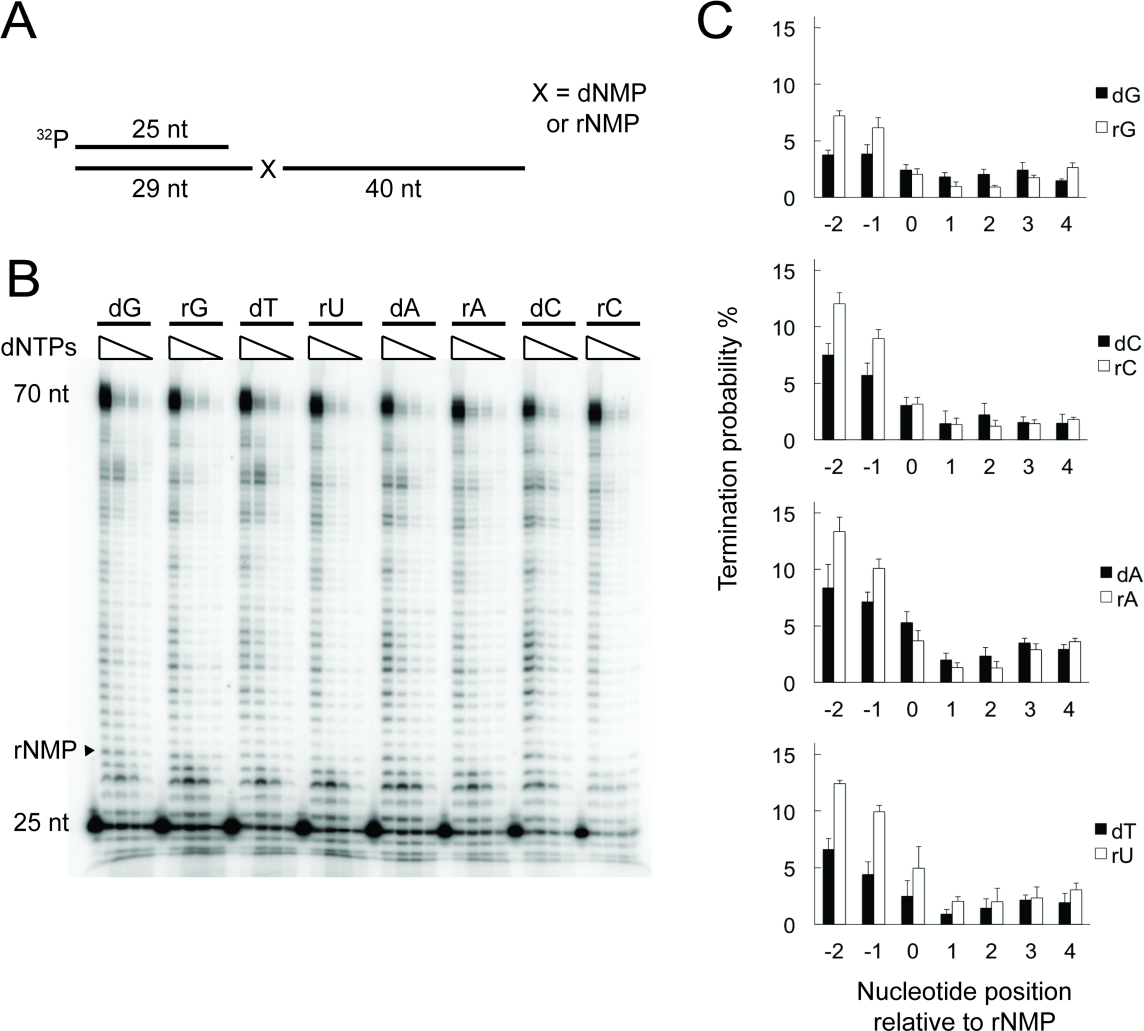
The termination probability of Pol γ on rNTP-containing templates. (**A**) Schematic diagram of the DNA substrate used in the Pol γ polymerase activity assay in Fig 1B. The substrate consisted of a 5′-labelled 25-nucleotide primer annealed to a 70-mer template strand containing either a dNMP or a rNMP (indicated with “X”) at the +5 position relative to start of DNA synthesis. (**B**) DNA polymerase activity of wild type Pol γ on rNMP-containing templates (rG, rU, rA, rC) and dNMP-containing control templates (dG, dT, dA, dC). The concentrations of dNTPs were 1 μM, 0.1 μM, 0.05 μM and 0.01 μM in single rNMP bypass conditions. The gel is a representative picture of three independent experiments. (**C**) Termination probabilities (y-axis) at positions surrounding a single template rNMP under conditions where the product is derived from a single round of synthesis (single-hit conditions) The DNA substrate was present at a 50-fold molar excess over Pol γ in order to minimize re-initiation of synthesis on the same template after an initial termination event. Bars represent the mean of three time points (2, 4 and 6 min) ± standard deviation.

Mammalian mtDNA is known to contain embedded rNMPs [10,13,24]. We confirmed the uniform distribution of rNMPs in mouse liver mtDNA by Southern blot analysis and found rNMPs to be embedded on average every 500 nucleotides on either strand (S1 Fig). Due to the relative frequency of rNMPs in mature mtDNA, Pol γ is expected to encounter several rNMPs in the template strand during replication of the ~16 kb mtDNA molecule. If these rNMPs impair replication, they could have pathological consequences for patients that, as a result of a shortage of specific dNTPs, have increased levels of rNMPs in mtDNA [10]. The exonuclease deficient variant of Pol γ has previously been reported to be able to bypass rNMPs [18], however, we wanted to examine the bypass of wild type Pol γ as well as the efficiency of bypass at a range of physiologically relevant dNTP concentrations. These *in vitro* polymerization reactions were carried out on synthetic DNA templates where Pol γ encountered the ribonucleotide at the 5^th^ position after the initiation of DNA synthesis (Fig 1A); the control templates contained the corresponding deoxyribonucleoside monophosphate (dNMP) in place of rNMP in an identical sequence context. We found that bypass of a single embedded rNMP by Pol γ was not obviously reduced relative to an all-dNMP template, even at the lowest dNTP concentration tested (0.01 μM; Fig 1B, compare dNMP/rNMP pairs). This result indicates that DNA synthesis by the Pol γ holoenzyme is not strongly inhibited by a single rNMP in the DNA template at the range of dNTP concentrations present *in vivo*. Similar results were observed with the exonuclease-deficient D274A Pol γ variant (S2 Fig).

The above reactions were performed with a 2.5-fold excess of polymerase over DNA template, whereby re-initiation of DNA synthesis could mask a moderate reduction in bypass efficiency. We therefore performed primer extension assays in reaction mixtures containing a large excess of template-primer over DNA polymerase, so that once a primer is extended, the probability that it will be used a second time is negligible, and the products therefore derive from a single cycle of synthesis. Band intensities were used to calculate termination probabilities at specific nucleotide positions surrounding the rNMP, as previously described ([25], also see Materials and Methods). Under these conditions, the presence of rNMP in the template led to a relatively moderate increase in termination probability, especially at positions -2 and -1 relative to the embedded rNMP (Fig 1C). In addition, the rUMP-containing template showed an increase in termination probability at position +1.

Taken together, the results of Fig 1 show that even at low dNTP concentrations the efficiency of Pol γ is only slightly affected by single rNMPs present in the DNA template, which is in agreement with the reverse transcriptase activity of Pol γ [26,27]. Notably, the observed effect of single rNMPs on the termination probability of Pol γ is considerably lower than that reported for yeast and human nuclear replicative DNA polymerases [28–30].

### Template rNMPs do not affect the fidelity of Pol γ

We next addressed the fidelity of rNMP bypass by sequencing the DNA products of a primer extension assay in order to determine the base inserted by Pol γ opposite a single template rNMP present at position +5 relative to the primer terminus. Sequencing analysis revealed that Pol γ incorporates the correct base opposite a rNMP with similar fidelity as opposite a dNMP (98.3% *versus* 98.2%, which are expected values for this PCR based assay). This shows that a single rNMP in the template has no detectable adverse effects on insertion fidelity. In contrast, Pol γ incorporated the correct base, dC, opposite 8-oxo-7,8-dihydroguanine (8-oxo-G) in only 68% of the sequenced products, which is comparable to the 73% found in an earlier report [31], validating the experimental set-up. The fidelity of the proofreading-deficient D274A mutant of Pol γ was found to be slightly lower opposite an rNMP (correct base in 96.4% of products), but a similar drop in fidelity was observed on an all-dNMP template (96.0%). These data suggest that unrepaired rNMPs in the mitochondrial genome are well-tolerated by Pol γ, not only when it comes to bypass efficiency (Fig 1), but also in terms of replication fidelity.

### Polymerase γ exhibits an unexpectedly low frequency of rNMP incorporation

**Fig 2:**
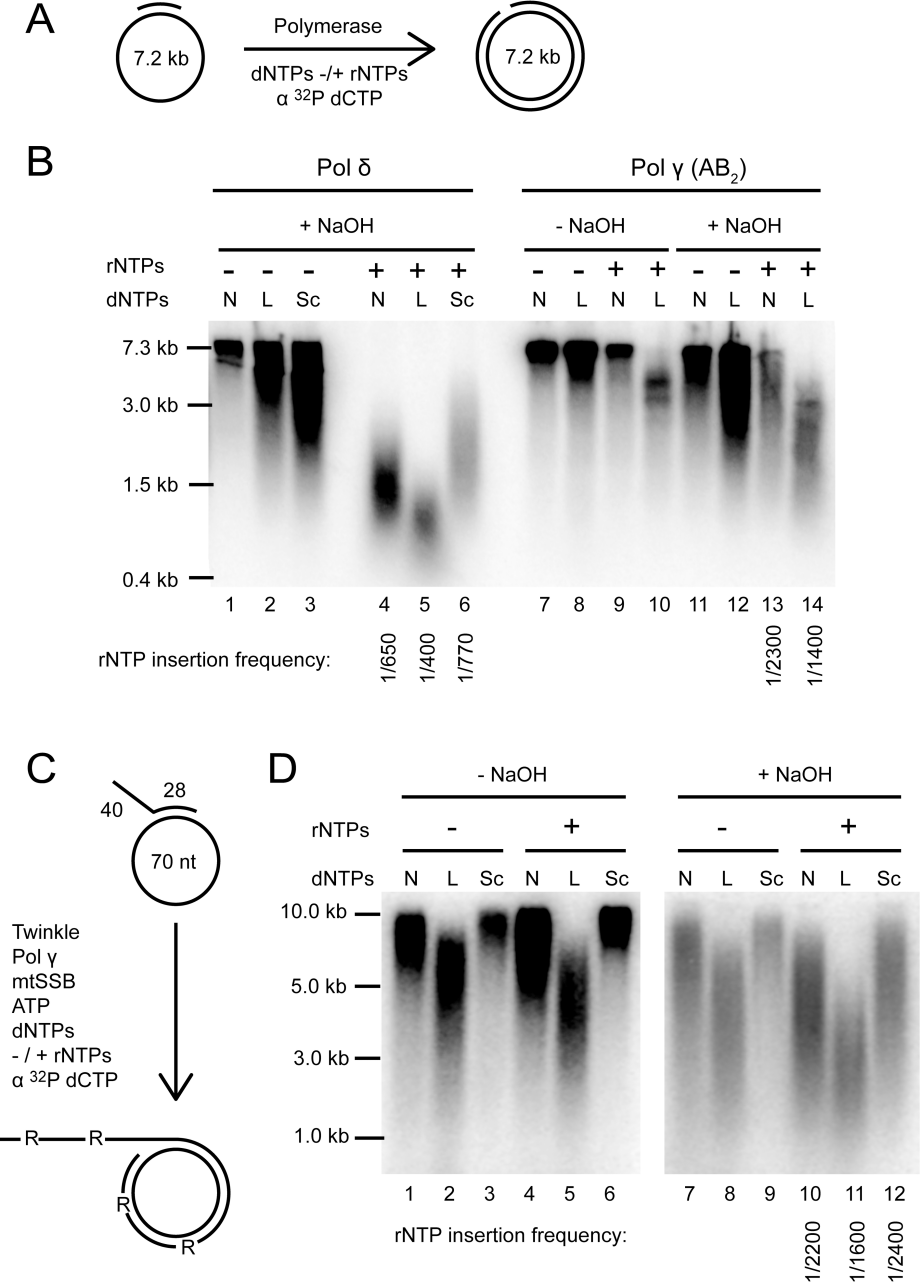
rNMP incorporation frequency of Pol γ and the mtDNA replisome on long DNA templates. (**A**) Schematic diagram of the 7.3 kb M13 ssDNA substrate used to compare the incorporation of rNMPs by Pol γ and yeast Pol δ in the primer extension assay in Fig 2B. The newly synthesized DNA was labelled by addition of [α-^32^P]-dCTP to the reaction. (**B**) Analysis of the rNMP incorporation frequency of Pol γ and yeast Pol δ at “normal” (“N”, S phase) concentrations, “low” (“L”, concentration during the rest of the cell cycle and in non-dividing cells), or *S*. *cerevisiae* (“Sc”) dNTP concentrations, in the absence or presence of rNTPs. In all reactions, the DNA template was coated with the relevant single-stranded DNA-binding proteins (lanes 1–6 with RPA; lanes 7–14 with mtSSB) to avoid stalling of DNA synthesis due to formation of DNA secondary structures. Untreated (“-NaOH”) or alkaline (“+NaOH”) treated reaction products were analysed on an agarose gel under denaturing conditions. To estimate rNMP incorporation frequencies from the presented gel, the median length of alkali stable products was determined and used to calculate the frequencies as described in Materials and Methods. Numbers on the left-hand side of the gel indicate positions of DNA marker bands and the full-length starting product (7.3) in kb. The gel is a representative picture of four independent experiments. (**C**) Schematic diagram of the primed mini-circle substrate with a 40 nt 5′ overhang used in Fig 2D. (**D**) Analysis of the rNMP incorporation frequency by the mitochondrial replisome consisting of mtSSB, Twinkle and Pol γ (AB_2_) on a primed mini-circle substrate with a 5′ overhang for Twinkle loading. Reactions were carried out at normal (“N), low (“L”), and “*S*. *cerevisiae*” (“Sc”) dNTP concentrations in the presence or absence of rNTPs. Untreated (“-NaOH”) and alkaline (“+NaOH”) treated reaction products were analysed on a denaturing (alkaline) agarose gel. The gel is a representative picture of two independent experiments.

Pol γ, the replicative mitochondrial polymerase, has been suggested to be the main source of the rNMPs embedded in the mitochondrial genome, and it has been shown to incorporate one rNMP for every 2.0 ± 0.6 × 10^3^ bases on a short 70 nt template *in vitro* [10]. However, earlier work on nuclear DNA polymerases has shown that *in vitro* analysis of rNMP insertion frequency is greatly affected by the sequence context of the short, defined oligonucleotides used in these studies [32]. To circumvent this problem, we used the 7.3 kb M13 ssDNA as a template to estimate the propensity of Pol γ to incorporate rNMPs (Fig 2A). Use of a long DNA template allows many sequence contexts to be tested simultaneously and the results should therefore give a closer estimate of the rNMP incorporation frequency occurring *in vivo*. To further increase the relevance of our data, we aimed to perform the reactions at physiologically relevant rNTP and dNTP concentrations. In an attempt to simulate the conditions that prevail during different stages of the cell cycle, two different sets of dNTP concentrations were tested: “normal” that mimic the concentrations present during S phase, and “low” that represent concentrations found during the rest of the cell cycle [33–35]. The latter dNTP concentrations are also expected to be similar to those found in non-dividing cells where nuclear DNA is not being replicated, but mtDNA replication still occurs. As a reference, we carried out reactions using the *S*. *cerevisiae* lagging strand DNA polymerase, Pol δ, of which the rNMP incorporation frequency has been reported using an identical assay set-up [7]. Unfortunately, comparison to human nuclear replicative polymerases is prevented by the lack of data on their rNMP incorporation frequencies using a similar long-template assay, and the general discrepancy between incorporation frequencies determined using long *vs*. short templates [7]. To enable comparison to literature values, some reactions with Pol γ and yeast Pol δ were performed using the nucleotide concentrations measured from logarithmically growing *S*. *cerevisiae* cells [2]. In all reactions, the DNA template was coated with the relevant single-stranded DNA binding protein (Fig 2B, lanes 1–6 with RPA; lanes 7–14 with mtSSB) to avoid stalling of DNA synthesis due to the formation of secondary DNA structures.

Radioactively labelled products of the primer extension reactions on M13 ssDNA were treated with NaOH to hydrolyse the phosphodiester bond on the 3′ side of incorporated rNMPs and analysed by agarose gel electrophoresis under mildly denaturing conditions (Fig 2B). In the absence of rNTPs, Pol γ synthesized long products that were only moderately affected by alkaline treatment (Fig 2B, compare lanes 7–8 and 11–12). In contrast, the DNA products synthesized in the presence of rNMPs were alkali-sensitive and a range of smaller DNA products was observed (Fig 2B, compare lanes 9–10 and 13–14) indicating rNMP incorporation. The distribution of radioactive signal in individual lanes was quantified and transformed to a size distribution as previously described [7] in order to determine the median length of alkali-stable DNA fragments. The median length values were further used to determine the frequency of rNMP incorporation (see Materials and Methods for details). Please note that because the signal in Fig 2B derives from incorporation of α-^32^P dCTP, longer products that contain a larger number of radioactive nucleotides appear stronger in intensity than shorter products. This causes the apparent product length on the gel to appear longer than the actual median length determined from the size distribution plot (in which the signal has been corrected for length). At “normal” dNTP levels, the median length of untreated DNA products synthesized by Pol γ was 5.2 kb (Fig 2B, lane 9) and this value dropped to 1.6 kb after NaOH treatment (Fig 2B, lane 13). These median lengths correspond to an average rNMP incorporation frequency of 1 rNMP per 2300 nt at “normal” dNTPs levels, while at “low” dNTP concentrations, the average rNMP incorporation frequency was 1 rNMP per 1400 nt (Fig 2B, lane 10 *vs*. lane 14). The calculations take into account that Pol γ was unable to fully replicate the 7.3 kb template in the presence of “low” dNTPs (Fig 2B, lane 10) due to a strong reduction in replication rate in the presence of rNTPs.

In comparison, the rNMP incorporation frequencies obtained for Pol δ were 1 rNMP per 770, 650 and 400 nt at “*S*. *cerevisiae”*, “normal” and “low” dNTP concentrations, respectively (Fig 2B, lanes 1–6). The rNMP incorporation frequency at “*S*. *cerevisiae”* dNTP concentrations is in excellent agreement with the previously reported value of 1 rNMP per 720 nts obtained using an identical approach [7], thus validating our experimental set-up. In conclusion, the results of Fig 2B show that Pol γ incorporates rNMPs far less frequently than its yeast homolog Mip1 (1 rNMP per 600 nts; [11]) or the yeast nuclear replicative polymerases Pol δ or Pol ε (1 rNMP per 720 or 640 nts, respectively [7]).

### rNMP incorporation frequency by the human mitochondrial replisome

For processive replication of double-stranded mtDNA, Pol γ requires the activity of the mitochondrial DNA helicase Twinkle. To examine the impact of Twinkle on rNMP incorporation by Pol γ, we constructed a DNA substrate consisting of a primed single-stranded mini-circle with a 40 nt 5′-overhang to allow Twinkle loading (Fig 2C). Once initiated, leading-strand DNA synthesis, coupled to continuous unwinding of the DNA template, can in theory continue indefinitely (rolling circle replication). This replication system requires ATP, since DNA unwinding by Twinkle is an ATP-dependent process. As the concentration of ATP normally employed in our *in vitro* assays (4 mM) would interfere with the rNMP incorporation studies, we made use of a creatine phosphokinase-based ATP regeneration system that maintained the ATP concentration at a lower concentration of approximately 300 μM. This way, the influence of free ATP in the assays without rNTPs was minimized while ensuring that efficient rolling circle replication could occur (S3 Fig).

In the absence of rNTPs, the reconstituted human mitochondrial replisome consisting of Pol γ, Twinkle and mtSSB synthesized long DNA fragments at all dNTP concentrations tested (Fig 2D, lanes 1–3). Alkali treatment of the reactions lacking rNTPs resulted in a small drop in DNA fragment size, which was ascribed to the presence of the 300 μM ATP required for Twinkle function (Fig 2D, compare lanes 1–3 with lanes 7–9). In contrast, the DNA products synthesized in the presence of rNTPs showed a substantial reduction in length upon alkali treatment, consistent with rNTP incorporation (Fig 2D, compare lanes 4–6 with lanes 10–12). The median length of DNA products synthesized in the presence of rNTPs was 5.2 kb using “normal” and 1.9 kb using “low” dNTP concentrations (Fig 2D, lane 4 and 5). NaOH treatment reduced the median length of the DNA products to 1.5 kb and 0.86 kb under “normal” and “low” dNTPs concentrations, respectively (Fig 2D, lane 10 and 11). Based on these median length values, the average rNTP insertion frequency of Pol γ holoenzyme in the presence of the helicase Twinkle and mtSSB is 1 rNMP per 2200 and 1600 nts at “normal” and “low” nucleotide concentrations, respectively. As expected, at the somewhat higher dNTP concentrations prevailing in logarithmical-growing *S*. *cerevisiae* cells, the rNMP incorporation frequency was the lowest, 1 rNMP per 2400 nts. These incorporation frequencies are similar to the values obtained in the reactions without Twinkle (Fig 2B), showing that the rNMP incorporation propensity of Pol γ is not affected by the presence of other core mitochondrial replication factors.

### The exonuclease activity of Pol γ is unable to repair incorporated rNMPs

Numerous studies have shown that nuclear replicative DNA polymerases proofread incorporated rNMPs poorly, if at all [29,30,36]. Because proofreading of rNMPs could explain the relatively low rNMP incorporation frequency by Pol γ observed in Fig 2B, we tested the contribution of its exonuclease activity to rNMP incorporation both *in vitro* and *in vivo*.

**Fig 3:**
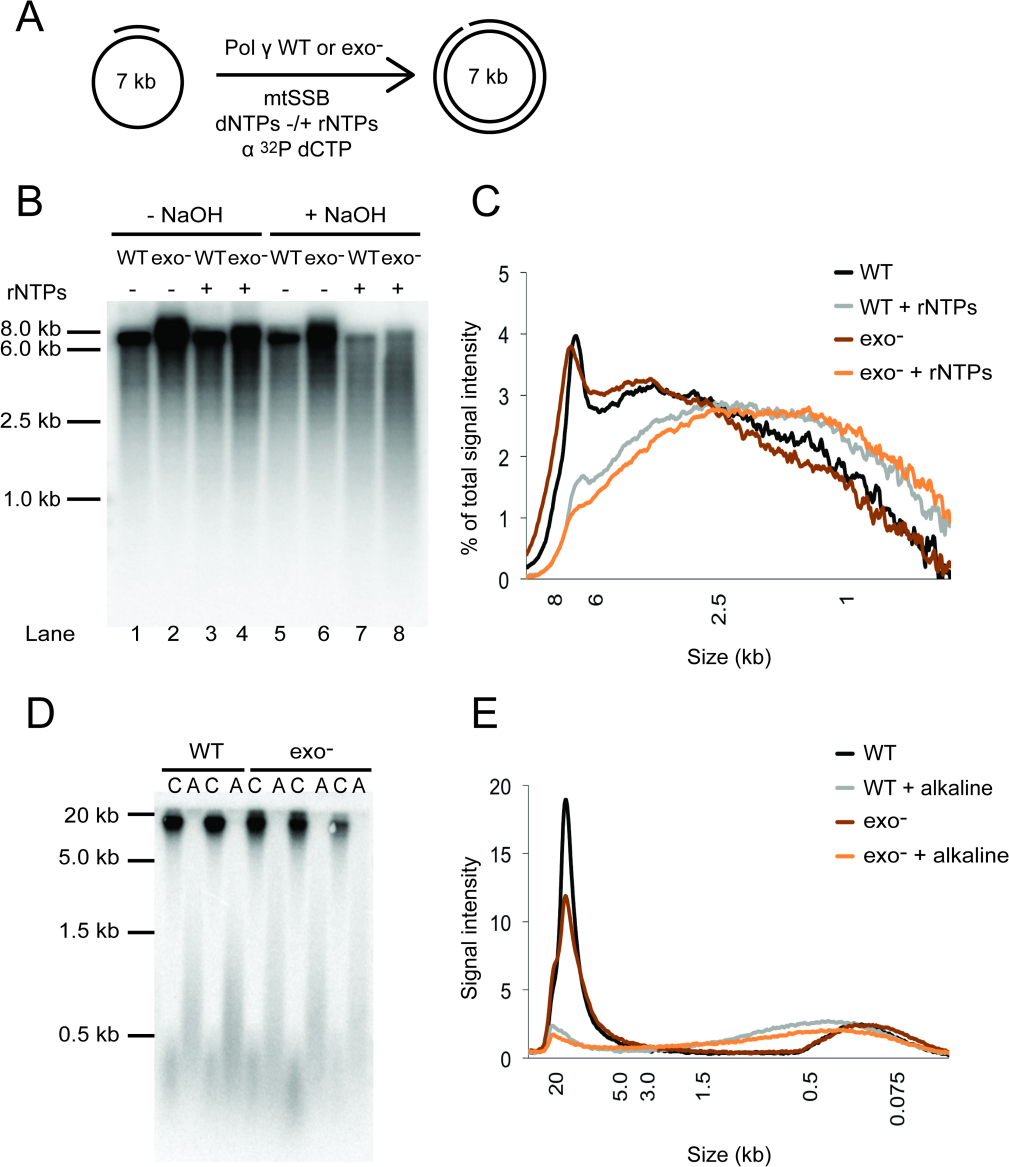
The influence of exonuclease activity on rNMP incorporation by Pol γ. (**A**) Schematic view of reaction set up in Fig 3B. (**B**) Comparison of rNMP incorporation frequencies of wild type (WT) and exonuclease deficient (exo^-^) Pol γ. *In vitro* replication of a primed 7.3 kb ssDNA template was performed with 10 μM dNTPs in the presence (+) or absence (-) of rNTPs. Reaction products were followed by addition of [α-^32^P]-dCTP. Samples were alkaline-treated (“+ NaOH” lanes 5–8) or untreated control (“-NaOH” lanes 1–4) for 2 h at 55°C and run on a denaturing alkaline gel. Fig 3B is a representative picture of three independent experiments. (**C**) Distribution plot of the percentage of total signal intensity from NaOH-treated samples in Fig 3B. The curves for WT (black and grey) and exo^-^ (brown and orange) Pol γ overlap, which indicates a similar incorporation frequency. (**D**) Southern blot analysis against the 16S rDNA region of mtDNA isolated from the liver of WT *PolgA* (n = 2) and exonuclease-deficient *PolgA*^*D257A*^ (n = 3; “exo^-^”) mice. SacI-linearized mtDNA was treated with alkaline hydrolysis (“A”) and run on an alkaline gel alongside untreated control samples (“C”). (**E**) Distribution plot of the DNA fragments in control and alkaline-treated samples from Fig 3D. The comparable distribution of DNA fragment size after alkaline-treatment is consistent with a comparable rNMP incorporation frequency in liver mtDNA of WT *PolgA* and *PolgA*^*D257A*^ mice.

First, we carried out *in vitro* primer extension assays on M13mp18 ssDNA with either wild type (WT) or exonuclease-deficient (exo^-^) Pol γ (Fig 3A). An extended reaction time (120 min) and addition of extra polymerase after half the time ensured that the majority of DNA products synthesized were full-length (7.3 kb) (Fig 3B, lanes 1–4). Analysis of NaOH-treated products synthesized in the presence of rNTPs revealed a comparable distribution of DNA fragment sizes from reactions catalysed by wild type or exo^-^ Pol γ (Fig 3B, lane 7 *vs*. 8; and Fig 3C, “WT + rNTPs” *vs* “exo^-^ + rNTPs”). This observation demonstrates that the proofreading activity of Pol γ does not influence the frequency with which it incorporates rNMPs *in vitro*.

To confirm our *in vitro* findings, we next isolated mtDNA from mice homozygous for an exonuclease-deficient mutant of the catalytic subunit of Pol γ (*PolgA*^*D257A /D257A*^, hereafter referred to as *PolgA*^*D275A*^). Due to the lack of proofreading activity by the PolgA^D275A^ variant, these mice accumulate mtDNA mutations and show signs of premature ageing [37]. Linearized mtDNA isolated from the liver of WT *PolgA*^*+/+*^ and the proofreading-deficient *PolgA*^*D275A*^ mutant mice migrated as expected as a 16 kb mtDNA molecule (Fig 3D, lanes denoted with “C”). Consistent with the results shown in S1C Fig, alkali treatment resulted in shorter DNA fragments (Fig 3D, lanes denoted with “A”). The size distribution plots of alkali-treated wild type and *PolgA*^*D275A*^ mtDNA were indistinguishable from each other, indicative of a comparable rNMP incorporation profile (Fig 3E). The results of the *in vivo* and *in vitro* experiments therefore both indicate that the exonuclease activity of Pol γ does not influence the amount of rNMPs embedded in the mtDNA.

### The presence of free rNTPs forces Pol γ into a polymerase/exonuclease idling mode, reducing replication speed

**Fig 4:**
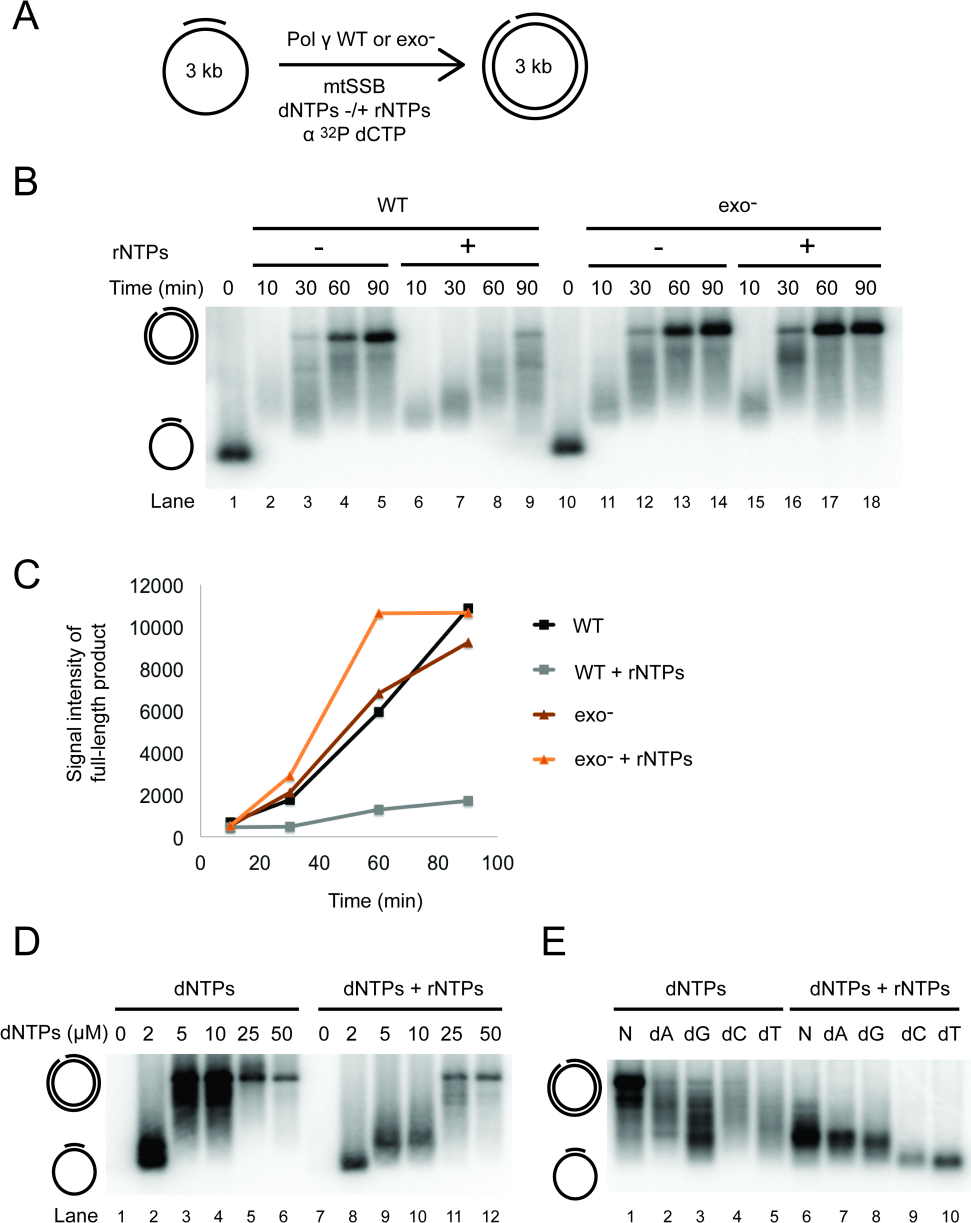
The effect of free rNTPs on the processivity of WT and exo^-^ Pol γ. (**A**) Schematic diagram of the processivity assay carried out with WT and exo^-^ Pol γ on a primed, circular 3 kb template in the presence and absence of rNTPs. (**B**) Analysis of the processivity of WT and exo^-^ Pol γ as depicted in Fig 4A. Reactions were stopped after 10, 30, 60, and 90 min and the DNA products were analysed by agarose gel electrophoresis. All reactions were carried out in the presence of 750 nM mtSSB and 10 μM dNTPs. Where indicated, rNTPs were added. Fig 4B shows a representative figure of five (WT) and three (exo^-^) independent experiments. See also S4 Fig. (**C**) A plot of the signal intensity of the full-length product at each time point in Fig 4B. (**D**) Processivity was tested during an increasing amount of dNTPs (0, 2, 5, 10, 25 or 50 μM) with or without rNTPs at fixed concentration. Reactions were stopped after 60 min and analysed on TBE agarose gel. (**E**) To stimulate the conditions of imbalanced dNTP pools, each dNTP was limited to 1 μM (indicated dA, dG, dC or dT) and compared with “normal” dNTP conditions (indicated as N). Reactions run for 60 min, with or without rNTPs present and analysed on TBE agarose gel.

Our findings presented so far indicate that rNMPs incorporated in the DNA template do not constitute a considerable impediment to replication by Pol γ. On the other hand, the lower amount of synthesis products observed in experiment in Fig 2B (lanes 7–10) suggested that the presence of free rNTPs in the reaction mix in addition to dNTPs may have an adverse effect on DNA synthesis by Pol γ *in vitro*, especially when dNTP concentrations are low. To better study the effect rNTPs on the rate of replication by Pol γ, we performed DNA replication experiments with the WT and exo^-^ Pol γ variants in the presence and absence of rNTPs using the 3 kb pBluescript DNA template (Fig 4A). As the reaction progressed, both the WT and exo^-^ Pol γ variants were able to efficiently produce a full-length 3 kb DNA product when only dNTPs were present in the reaction mix (Fig 4B, lanes 2–5 and lanes 11–14). However, the addition of rNTPs into the reaction substantially reduced the efficiency of replication catalysed by WT Pol γ, and only a faint full-length band was visible even after a 90-min reaction (Fig 4B, lanes 6–9). In contrast, the length of DNA products synthesized by the exo^-^ Pol γ variant was unaffected by the presence of rNTPs in the reaction (Fig 4B, compare lanes 11–14 with lanes 15–18). Quantification of full-length reaction products confirmed that the processivity of Pol γ was only affected by the presence of free rNTPs when its proofreading ability was intact (Fig 4C). This suggests that the presence of rNTPs forces the wild type enzyme to slow down and/or stall in a manner that depends on a functional exonuclease domain. However, as shown in Fig 3, the proofreading activity of Pol γ is unable to selectively remove incorporated rNMPs. Taken together, these findings are consistent with a scenario where the presence of free rNTPs forces WT Pol γ to idle between polymerase and exonuclease modes, leading to slower replication.

The above reactions were performed in the presence of 10 μM equimolar dNTPs. However, the rNTP-dependent decrease in Pol γ replication speed was even more pronounced at physiologically relevant (“normal”) dNTP concentrations (S4A Fig, lanes 6–9). Similar reactions with the *S*.*cerevisiae* homolog of Pol γ, Mip1, showed that also the replication rate of this polymerase was affected by the presence of the rNTPs, albeit to a lesser extent than its human counterpart (S4B Fig). Taken together, these results indicate that free ribonucleotides can impair the activity of mitochondrial polymerases, with human Pol γ being more affected than its yeast homolog Mip1.

### A high rNTP/dNTP ratio reduces mitochondrial replication speed

We next compared the effect of different dNTP concentrations on synthesis by Pol γ when rNTPs were present at constant, physiological concentrations. At high dNTP concentrations (25 and 50 μM), there was no striking difference in the size of the products from reactions carried out in the absence or presence of rNTPs (Fig 4D, compare lanes 5–6 with lanes 11–12). However, as dNTP levels decreased, the adverse effect of rNTPs on replication became increasingly evident. At 10 μM dNTPs, approximately half of the products of the dNTP-only reaction were full-length, while the rNTP-containing reactions yielded no full-length product (Fig 4D, lanes 4 and 10). Therefore, the apparent inhibition of synthesis by Pol γ in the presence of rNTPs was especially pronounced at low dNTP concentrations.

Finally, we simulated conditions of imbalanced dNTP pools by lowering one dNTP at a time to 1 μM, while the other dNTPs were kept at “normal” concentrations. As expected, limiting the concentration of one dNTP led to a visible reduction in the size of reaction products (Fig 4E, compare lane 1 to lanes 2–5 and lane 6 to lanes 7–10). However, while the reactions containing only dNTPs still yielded fairly long products (Fig 4E, lanes 1–5), the size of the rNTP-containing reaction products was far below full-length (Fig 4E, lanes 6–10). Decreasing the concentrations of dCTP or dTTP had a more striking effect than limiting dATP or dGTP. The reason that limiting pyrimidines had a greater effect than limiting purines is unclear, and could not be explained by sequence bias in the DNA template where all four bases are represented at equal frequencies. Taken together, the data in Fig 4 suggest that the combined effect of a limiting dNTP together with the presence of rNTPs can lead to severe stalling of replication by Pol γ.

## Discussion

The observation that mtDNA is rich in rNMPs has intrigued researchers for over four decades [10,13,18,24,38]. Although rNMPs are erroneously incorporated during DNA synthesis both in the nucleus and in mitochondria, RER efficiently removes the incorporated rNMPs from nuclear DNA. Unlike the nucleus, mitochondria appear to lack rNMP removal pathways [5,10], meaning that incorporated rNMPs persist and could therefore potentially interfere with mtDNA replication, although this phenomenon has not been studied in detail.

Our analysis of mouse mtDNA fragmentation by alkaline and RNase H2-treatment confirmed the relative abundance of embedded rNMPs in the mammalian mitochondrial genome, as well as their uniform distribution (S1 Fig). However, we show that the presence of rNMPs in the replication template does not negatively impact the efficiency or fidelity of replication by Pol γ, even at the lower dNTP concentrations that mimic the conditions in cycling or non-dividing cells (Fig 1). Therefore, the 3- to 4-fold increase in mtDNA rNMPs that was recently reported in fibroblasts derived from patients with disturbed mitochondrial dNTPs pools [10] is, based on our experiments, unlikely to be problematic for the mtDNA replication fork thanks to the high fidelity and efficiency of Pol γ when bypassing single embedded rNMPs.

Interestingly, we found that the frequency of rNMP incorporation by Pol γ was at least three-fold lower than that of the yeast nuclear Pol δ and Pol ε (Fig 2 and [7]). Based on our *in vitro* data, Pol γ is expected to incorporate about 14 rNMPs during the synthesis of one 16.5 kb dsDNA molecule of mtDNA in cycling cells (Fig 2B lane 13). This value is likely to be higher (24 rNMPs) in post-mitotic cells in which the dNTP levels are thought to be substantially lower (Fig 2B lane 14). However, our Southern blot analysis of mouse liver mtDNA indicated an *in vivo* rNMP frequency of approximately 1 rNMP per 500 nucleotides (S1 Fig; corresponds to 65 rNMPs per ds mtDNA), which is in good agreement with the values recently reported using a genome-wide next-generation sequencing approach (54 rNMPs per mtDNA molecule in human fibroblasts and 36 in HeLa; *i*.*e*. a frequency of 1:613 and 1:920, respectively [10]). The *in vivo* rNMP frequency of mtDNA is therefore 2- to 5-fold higher than expected from the rNMP incorporation rates observed in our *in vitro* experiments (Fig 2B and 2D). This difference may partly be explained by lower than estimated dNTP concentrations or higher than estimated rNTP concentrations inside mitochondria. We found that decreasing dNTP concentrations to as low as 0.5–1 μM in the *in vitro* replication assay gave rise to an rNMP incorporation frequency that is comparable to the *in vivo* frequency (1:620; S5B Fig) using exo^-^ Pol γ. However, such low dNTP concentrations in the presence of rNTPs do not support DNA synthesis by the WT Pol γ enzyme (S4A Fig lane 6–9) and are therefore not likely to be the sole explanation to the higher *in vivo* rNMP frequencies observed by us and others. An alternative, or contributing, explanation for the discrepancy between *in vivo* and *in vitro* rNMP levels is that additional polymerases in addition to Pol γ could contribute to incorporation of rNMPs into mtDNA, which then persist due to the absence of ribonucleotide excision repair inside mitochondria [10,11]. For instance, Pol β was recently detected in mitochondria [39]. Interestingly, Pol β levels can greatly affect rNMP incorporation in nDNA opposite oxidative DNA lesions [40]. Given the presumably high levels of oxidatively damaged nucleotides in mtDNA [41], incorporation by Pol β could significantly contribute also to rNMP incorporation frequencies in mtDNA. Similarly, the primase/polymerase PrimPol can synthesize nucleic acids using both rNTPs and dNTPs, and has been shown to be involved in mtDNA maintenance [42–44]. Together, these and possibly other polymerases could influence the rNMP levels in mtDNA [45].

We conclusively show that the comparably low frequency of rNMP incorporation by Pol γ is not due to efficient proofreading of rNMPs, as liver mtDNA from WT and proofreading-deficient Pol γ mice exhibited an indistinguishable profile and frequency of incorporated rNMPs (Fig 3D and 3E). Furthermore, *in vitro* incorporation frequencies were similar for WT and exonuclease-deficient Pol γ (Fig 3B and 3C). Therefore, the low rNMP frequency of Pol γ is expected to be due to efficient discrimination against rNTPs during the insertion step of DNA synthesis.

Finally, we find that the presence of free rNTPs significantly decreases the replication speed by Pol γ (Fig 4). A similar phenomenon has been described for family B polymerases [7,46], but to our knowledge, this is the first report of such inhibition in a family A polymerase. The negative effect of rNTPs on replication by Pol γ is dependent on the proofreading-activity of the polymerase and is therefore likely due to idling between the polymerase/exonuclease modes in the presence of rNTPs. The inhibition of Pol γ by rNTPs was most pronounced at low dNTP concentrations; at 10 μM dNTPs, a concentration that is comparable to that found in cycling cells, the negative effect of rNTPs impeded synthesis of a full-length product in our assay (Fig 4D). It is likely that at high rNTP/dNTP ratios, Pol γ requires more time to find the correct dNTP as the abundant corresponding rNTP acts as a competitive inhibitor of the enzyme. We show that especially in combination with the decreased level of a single dNTP, the presence of rNTPs in the reaction causes severe replication stalling (Fig 4E). These findings lead us to speculate that the high rNTP/dNTP ratio normally found in cells can be especially challenging in combination with a dNTP pool imbalance such as the ones found in patients suffering from mutations in thymidine kinase 2 (TK2) or deoxyguanosine kinase (DGUOK). Frequent replication stalling under such conditions might prevent the mtDNA replisome from completing replication of the entire mitochondrial genome, potentially leading to mtDNA depletion as it has been reported in patients affected by mtDNA depletion syndromes [47].

## Materials and methods

### Ethics statement

MtDNA mutator mouse samples were obtained from the Stewart lab at the Max Planck Institute for Biology of Ageing, Cologne Germany, where the mice are raised and handled in strict accordance to the guidelines of the Federation of European Laboratory Animal Science Associations (FELASA). Breeding and sacrifice protocols were approved by the “Landesamt für Natur, Umwelt und Verbraucherschutz Nordrhein-Westfalen" (84–02.04.2015 & 84–02.05.50.15.004)."

### Mouse strain

Wild type C57BL/6J mice were euthanized at the age of 2 months and livers were frozen in liquid nitrogen. Liver samples from mtDNA mutator mice were gifted from J.B. Stewart of the Max Planck Institute for Biology of Ageing, Cologne, Germany. These mice carried the PolgA^D257A^ allele [37], but were backcrossed onto the C57Bl/6J nuclear genetic background, and used to generate the mice for this study. Three homozygous mtDNA mutator mice (1 male, 2 females), and two wild type sibling controls (1 male, 1 female) at 50–53 weeks of age were used. Mice were bred to limit female-transmitted mtDNA mutations [48]. All animal procedures were conducted in accordance with European, national and institutional guidelines and protocols, and were approved by local government authorities.

### Isolation of mitochondrial DNA from mouse liver

Mouse liver was minced into pieces and homogenized using a glass teflon Dounce homogenizer in homogenization buffer (10 mM HEPES pH 7.8 with 225 mM mannitol, 75 mM sucrose and 10 mM EDTA) followed by centrifugation at 800 × g for 10 min at 4°C. The supernatant was then centrifuged at 12 000 × g for 10 min at 4°C to pellet mitochondria that were resuspended in homogenization buffer and overlaid on a 1.5 M/1M sucrose gradient in 10 mM HEPES pH 7.4 and 10 mM EDTA. After ultracentrifugation at 40 000 × g for 1 h at 4°C in a Beckman SW60Ti rotor, the mitochondrial layer was recovered and diluted in 4 volumes of 10 mM HEPES pH 7.4, 10 mM EDTA. The mitochondria were pelleted at 12 000 × g for 10 min at 4°C in a JA25.5 rotor, resuspended in homogenization buffer, treated with Proteinase K, lysed in 20 mM HEPES pH 7.8, 75 mM NaCl, 50 mM EDTA, 1% SDS and treated again with Proteinase K. The mtDNA was extracted once with (25:24:1) phenol:chloroform:isoamyl alcohol and twice with chloroform. The DNA was precipitated and resuspended in 20 mM HEPES pH 7.2.

### Southern blot analysis

1 μg of isolated mtDNA was linearized with SacI, precipitated and dissolved in 10 mM Tris HCl pH 7.5. The DNA was hydrolyzed with 0.3 M NaOH at 55°C or digested with RNase H2 (New England Biolabs) at 37°C for 2 h. Samples were run on a 0.8% agarose alkaline gel (30 mM NaOH, 1 mM EDTA) at 25 V, 4°C for 20 h and blotted onto Hybond-N+ membrane (Amersham, GE Healthcare). Single-stranded probes were end-labelled with γ-^32^P ATP using T4 polynucleotide kinase (Thermo Scientific) following the manufacturer’s protocol. Double-stranded probes were generated by labelling an approximately 500 bp PCR product with α-^32^P dCTP using Prime-It II Random Primer Labeling kit (Agilent Technologies). Hybridization was for 16 h at 42°C for ssDNA probes and at 65°C for dsDNA probes. The membrane was exposed to a PhosphoImager screen and scanned in a Typhoon laser scanner (GE Healthcare). The radioactive intensity was quantified using ImageJ software and plotted on a distribution plot. The median size of alkali-treated products was determined from the distribution of the radioactivity intensity and related to the size marker that was run in parallel [7].

### Purification of recombinant proteins

Human mitochondrial DNA polymerase γ catalytic subunit A (Pol γ A), processivity subunit B (Pol γ B), helicase Twinkle and mitochondrial single-stranded binding protein (mtSSB) were expressed and purified as previously described [49,50]. For the exonuclease-deficient Pol γ A, a D274A mutant was prepared as previously described [51]. *Saccharomyces cerevisiae* RPA [52], PCNA [53] and RFC [54] were purified from *Escherichia coli* overexpression systems, while *S*. *cerevisiae* Pol δ [55] was purified from a yeast overexpression system. Wild type *S*. *cerevisiae* mitochondrial DNA polymerase Mip1 was purified as previously described [11].

### DNA substrate preparation

Single-stranded M13mp18 DNA (7.3 kb) was purchased from New England Biolabs. Single-stranded pBlueScript SK+ was prepared as previously described [56]. Primers were either 5′-end labelled with γ-^32^P using T4 polynucleotide kinase (Thermo Scientific) or purchased with 5′-TET fluorescence label. A 36-mer primer 6330 (Figs 2B and 3B) or primer 682 (Fig 4 and S4 Fig) were annealed in a 1:1 ratio to M13mp18 or pBluescript SK+, respectively. For the linear 70 nt templates, a 25 nt primer (Fig 1B) was annealed to the template containing either a dNTP or a rNTP at position 30 (S1 Table) by heating to 80°C and slowly cooling to room temperature. The 70 nt mini-circle template (Fig 2C) was prepared as described in [51]. All oligonucleotides used in this study are listed in S1 Table.

### Incorporation and processivity assay

Primer extension was performed using 2.5–10 nM primed circular ssDNA with 12.5 nM of WT or exo^-^ Pol γ A and 18.75 nM of Pol γ B (as dimer). Additional protein was added after half the incubation time when indicated. MtSSB was added to a final concentration of 750 nM. The following reaction conditions were used: 25 mM Tris-HCl pH 7.6, 10 mM MgCl_2_, 1 mM DTT and 100 μg/ml BSA; dNTPs and/or rNTPs were added at indicated concentrations and run at 37°C. The reactions with Pol δ were performed essentially as described [7] at 30°C with the following protein concentrations: 3 nM Pol δ, 375 nM RPA, 15 nM PCNA (as trimer), and 3 nM of RFC. Mip1 reactions were performed with 5 nM of Mip1 in the same buffer conditions as Pol γ, but at 30°C. The reactions were performed in the presence of what we termed”normal” (5 μM dATP, 5 μM dCTP, 3 μM dGTP and 10 μM dTTP), “low” (2 μM dATP, 1 μM dCTP, 1 μM dGTP and 2 μM dTTP) or “very low” (1 μM dATP, 0.5 μM dCTP, 0.5 μM dGTP and 1 μM dTTP) dNTP concentrations. rNTP concentrations were kept constant (3 000 μM ATP and 500 μM of CTP, GTP and UTP). As the relatively high concentration of rNTPs could lead to sequestering of divalent cations, additional magnesium (4.5 mM) was included in the reactions containing rNTPs in order to maintain a constant concentration of magnesium throughout the experiment. As a reference, some reactions were performed with the concentrations of nucleotides found in logarithmically-growing *S*. *cerevisiae* cells: 16 μM dATP, 14 μM dCTP, 12 μM dGTP, 30 μM dTTP, 3 mM ATP, 0.5 mM CTP, 0.7 mM GTP and 1.7 mM UTP [2]. To follow the reaction, [α-^32^P]-dCTP (Perkin Elmer) was added. The reactions were incubated at 37°C for 1–120 min, stopped with 0.5% SDS, 25 mM EDTA and cleaned over G-25 columns to remove excess [α-^32^P]-dCTP. For incorporation assays, the sample was divided in two; one control was treated with 0.3 M NaCl and one sample was treated with 0.3 M NaOH. Before gel loading both samples were incubated for 2 h at 55°C. For use as a size reference, 1 kb GeneRuler (ThermoScientific) was end-labeled with γ-P^32^ ATP using T4 polynucleotide kinase (ThermoScientific). Samples and size marker were separated on a 1.5% alkaline agarose gel in buffer with 30 mM NaOH and 1 mM EDTA at 17 V for 16 h in 4°C. Visualization and quantification was performed by phosphoimaging of the dried gel on a Typhoon 9400 system (GE Healthcare).

### In vitro DNA synthesis with the mtDNA replisome

The mini-circle substrate (5 nM) was added to a 10 μl reaction mixture containing 25 mM Tris HCl pH 7.5, 75 mM NaCl, 10 mM magnesium acetate, 1 mM DTT, 100 μg/ml BSA, 4 mM ATP, 12.5 nM Pol γ A, 18.75 nM Pol γ B (as dimer), 250 nM mtSSB and 12.5 nM Twinkle. To keep the ATP concentration as low as possible in the reactions without rNTPs, we used an ATP regeneration system consisting of 400 ng creatine kinase and 5 mM creatine-phosphate-Tris. The reactions included the addition of “low”, “normal” or “*S*. *cerevisiae"* dNTPs (concentrations listed above) and rNTPs where indicated. The reactions were performed at 37°C and started by addition of polymerase. At the indicated time points, the reactions were stopped by the addition of 1.1 μl of termination mixture (5% SDS, 250 mM EDTA) and analyzed on an 8% polyacrylamide gel containing 8 M urea. Quantification was performed by phosphoimaging of the dried gel on a Typhoon 9400 system (GE Healthcare).

### Determination of average rNMP incorporation frequency

The average rNMP incorporation rate of Pol γ *in vitro* (Fig 2B and 2D) was determined as previously described [7]. Briefly, we determined the median size of DNA fragments in the reaction with only dNTPs (**a**) and in the reaction with both dNTPs and rNTPs (**b**). These values were used in the following formula to calculate the incorporation frequency: rNTP incorporation frequency = a/(a/b—1).

### Bypass of rNMPs

5–10 nM of template was used in primer extension reactions with 25 mM Tris HCl pH 7.5, 10 mM MgCl_2_, 1 mM DTT, 100 μg/ml BSA, 12.5 nM Pol γ A and 18.75 nM Pol γ B (calculated as dimer), with an increasing amount of dNTPs. For single hit conditions, reactions contained 50 nM template and 1 nM protein. Reactions were stopped after 2–20 min with 0.5% SDS, 25 mM EDTA and incubated at 50°C for 10 min. The samples were run on a 10–12% polyacrylamide urea gel, dried and exposed to a phosphoimager screen and scanned in a Typhoon 9400 system. Termination probability was calculated as previously described [25]. Briefly, at template position N, the termination probability was determined by the intensity of the band at N and divided by the intensity at position N plus the intensity at bands for longer products, at N, = [N] / ≥ [N].

### Sequencing of bypass products

The sequencing of *in vitro* synthesized DNA was performed as described [51] except that the primer extension reactions were performed on a 70 nt template containing either a dNMP or rNMP at position 30. As control template, a 70 nt oligo with 8-oxo-G at position 30 was used. The template was primed with a biotinylated oligonucleotide with a HindIII site (S1 Table). Reactions were stopped by incubation at 70°C for 1 h. Following the manufacturer’s instructions, the bypass products were immobilized on Dynabeads M-280 Streptavidin for 15 min at room temperature. The two strands were denaturated two times in 0.1 M NaOH for 5 min. The single-stranded product was washed according to the manufacturer’s instructions and amplified by high fidelity PCR using Phusion High-fidelity DNA polymerase (NEB) to generate a double-stranded 104 bp product. The fragment was cleaved using BfaI and HindIII restriction enzymes and cloned into pUC19. The ligation was transformed into *E*. *coli* TOP10 and colonies were sequenced with the M13 (-49) primer.

## Supporting information

S1 Fig The presence of rNMPs in mouse mtDNA. (**A**) Scheme of the experiment shown in S1C Fig. MtDNA linearized by SacI cleavage was treated with alkali or RNase H2 and run on a mildly denaturing agarose gel to examine the presence of incorporated rNMPs. Alkaline treatment and RNase H2 hydrolyse the phosphodiester bond adjacent to an embedded rNMP. (**B**) Localization of single-stranded DNA probes used in the southern blot analysis in S1C Fig. The SacI site that was used for linearization is indicated. (**C**) Southern blot analysis of SacI-linearized mouse liver mtDNA following alkaline hydrolysis (“A”) or RNase H2 (“R”) treatment for 4 mtDNA regions with ssDNA probes specific to the heavy (“H”) or the light (“L”) strand. Linearized, but further untreated mtDNA was run in parallel (lanes marked “C”). The median DNA fragment sizes determined from the alkali-treated products on this representative experiment are indicated below the gel. *The presence of excess rRNA prevented the measurement of the median length for the H-strand of the 16S region.
(TIF)

S2 Fig Bypass of single rNMPs by the exonuclease-deficient D274A variant of Pol γ. The template contained either an rNMP (rG, rC, rA, and rU) or a dNMP (dG, dC, dA, and dT) at position +5 from the primer end (indicated by an arrow). See Fig 1A for a schematic of the DNA substrate. The concentrations of dNTPs were 1 μM, 0.1 μM, 0.05 μM and 0.01 μM and reactions contained a 2.5-fold excess of DNA polymerase over DNA template. The gel shows a representative picture of two independent experiments.
(TIF)

S3 Fig Efficient replication of the mtDNA replisome at 300 μM ATP with the addition of a creatine phosphokinase-based ATP regeneration system. (**A**) Schematic diagram of the 70 nt ssDNA substrate used to compare the incorporation of rNMPs by Pol γ in the rolling circle replication assay presented in S3B Fig. Replication by the mitochondrial replisome consisting of mtSSB, Twinkle and Pol γ (AB2) on a primed mini-circle substrate with a 5’ overhang for Twinkle loading. (**B**) Replication in the presence (+) and absence (-) of creatine kinase (400 ng) and 5 mM creatine-phosphate-Tris with the indicated concentration of ATP. Replication products were analysed on a denaturing alkaline agarose gel.
(TIF)

S4 Fig Processivity of DNA polymerization by Pol γ and yeast mitochondrial DNA polymerase Mip1 in the presence of free rNTPs. (**A**) Processivity of WT and exo^-^ Pol γ in “normal” dNTP concentrations in the presence or absence of rNTPs. The 3 kb pBluescript DNA substrate shown in Fig 4A was used and reactions were stopped after the indicated reaction times and separated in an agarose gel electrophoresis. (**B**) Comparison of processivity by WT Pol γ and yeast mitochondrial DNA polymerase Mip1 on 3 kb pBluescript DNA template. The reactions contained 10 μM dNTP concentrations in the presence or absence of rNTPs. The reactions were stopped at indicated time points and run on an agarose gel. The figure shows a representative picture of two independent experiments.
(TIF)

S5 Fig rNMP incorporation frequency of exo^-^ Pol γ at very low dNTP concentrations. **(A)** A schematic overview of the incorporation assay shown in (B). **(B)** Analysis of rNMP incorporation frequency of exo^-^ Pol **γ** at very low dNTP concentrations (1 μM dATP, 0.5 μM dCTP, 0.5 μM dGTP and 1 μM dTTP) on a 3 kb template. The samples were alkaline treated (+NaOH) and analysed on a denaturing alkaline agarose gel. The rNMP incorporation frequency was determined from the median length of alkali stable products, for more details see Materials and Methods. The incorporation frequency is the average of two independent experiments.
(TIF)

S1 Table Oligonucleotides used in this study. (PDF)
