# Supplementary figures and images for: The presence of rNTPs decreases the speed of mitochondrial DNA replication
Source: PLoS Genet. 2018 Mar 30;14(3):e1007315. doi: 10.1371/journal.pgen.1007315 (PMC5895052; doi:10.1371/journal.pgen.1007315)

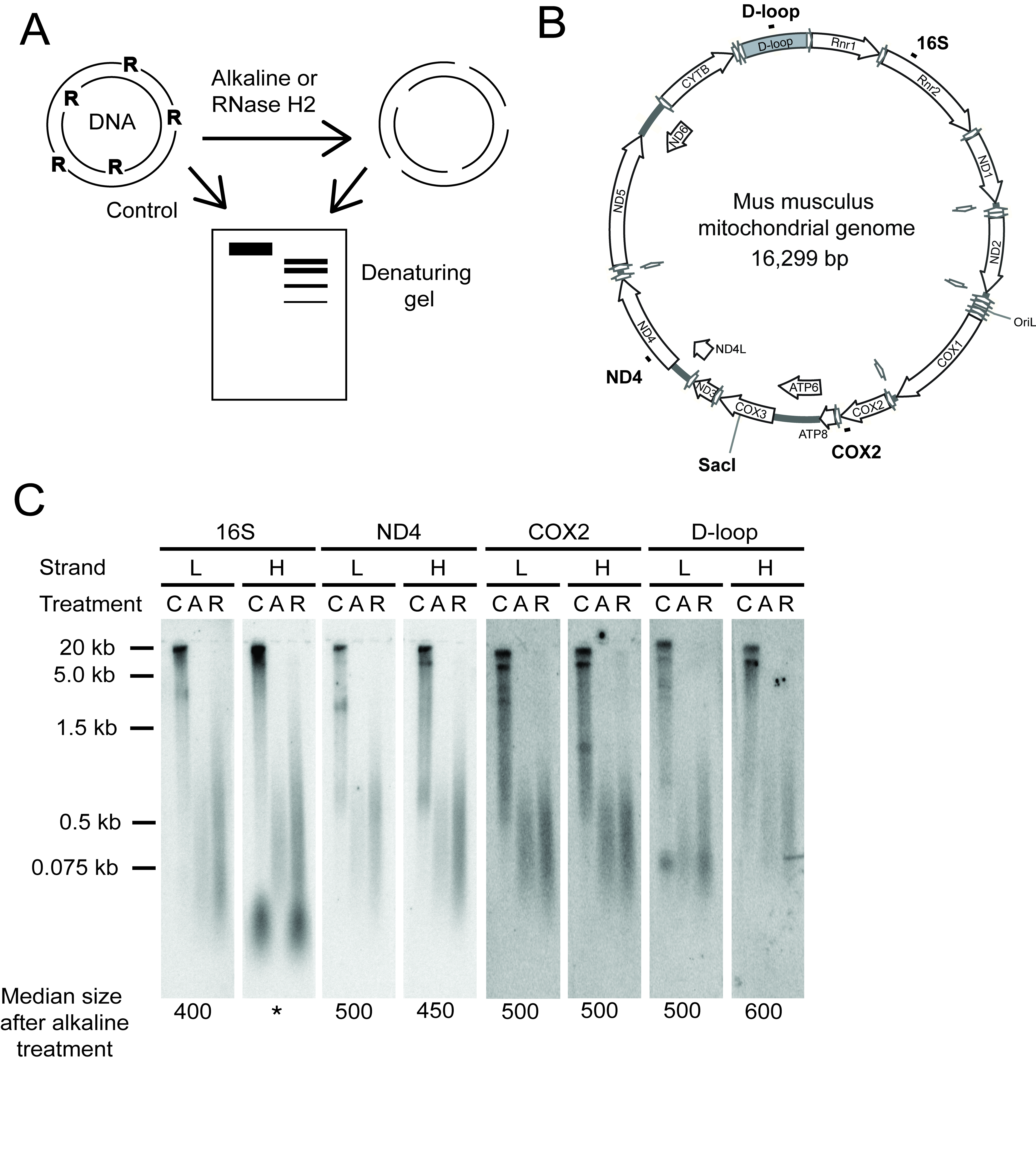

Supplement: S1 Fig — (A) Scheme of the experiment shown in S1C Fig. MtDNA linearized by SacI cleavage was treated with alkali or RNase H2 and run on a mildly denaturing agarose gel to examine the presence of incorporated rNMPs. Alkaline treatment and RNase H2 hydrolyse the phosphodiester bond adjacent to an embedded rNMP. (B) Localization of single-stranded DNA probes used in the southern blot analysis in S1C Fig. The SacI site that was used for linearization is indicated. (C) Southern blot analysis of SacI-linearized mouse liver mtDNA following alkaline hydrolysis (“A”) or RNase H2 (“R”) treatment for 4 mtDNA regions with ssDNA probes specific to the heavy (“H”) or the light (“L”) strand. Linearized, but further untreated mtDNA was run in parallel (lanes marked “C”). The median DNA fragment sizes determined from the alkali-treated products on this representative experiment are indicated below the gel. *The presence of excess rRNA prevented the measurement of the median length for the H-strand of the 16S region. (TIF) [file pgen.1007315.s001.tif]

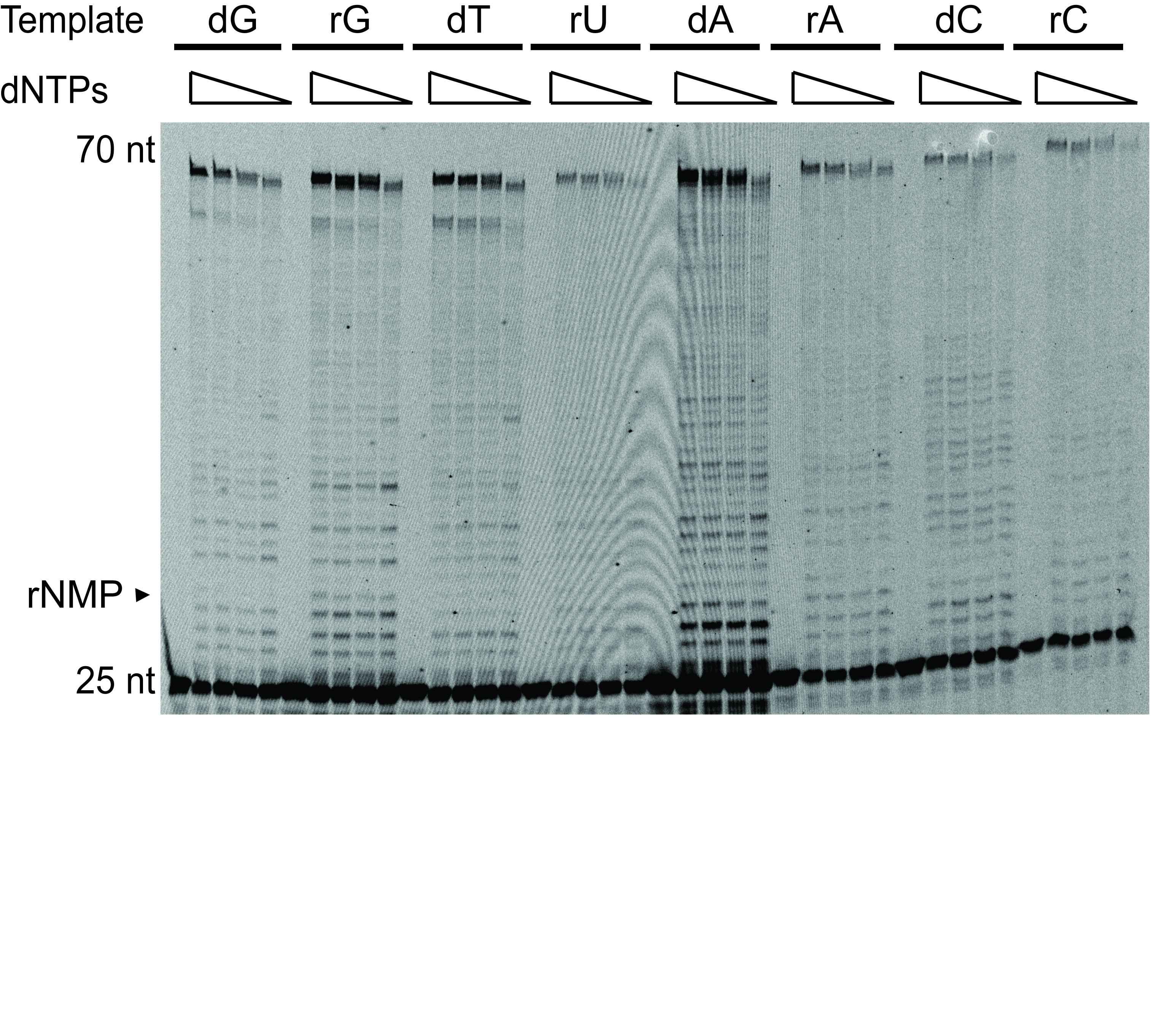

Supplement: S2 Fig — The template contained either an rNMP (rG, rC, rA, and rU) or a dNMP (dG, dC, dA, and dT) at position +5 from the primer end (indicated by an arrow). See Fig 1A for a schematic of the DNA substrate. The concentrations of dNTPs were 1 μM, 0.1 μM, 0.05 μM and 0.01 μM and reactions contained a 2.5-fold excess of DNA polymerase over DNA template. The gel shows a representative picture of two independent experiments. (TIF) [file pgen.1007315.s002.tif]

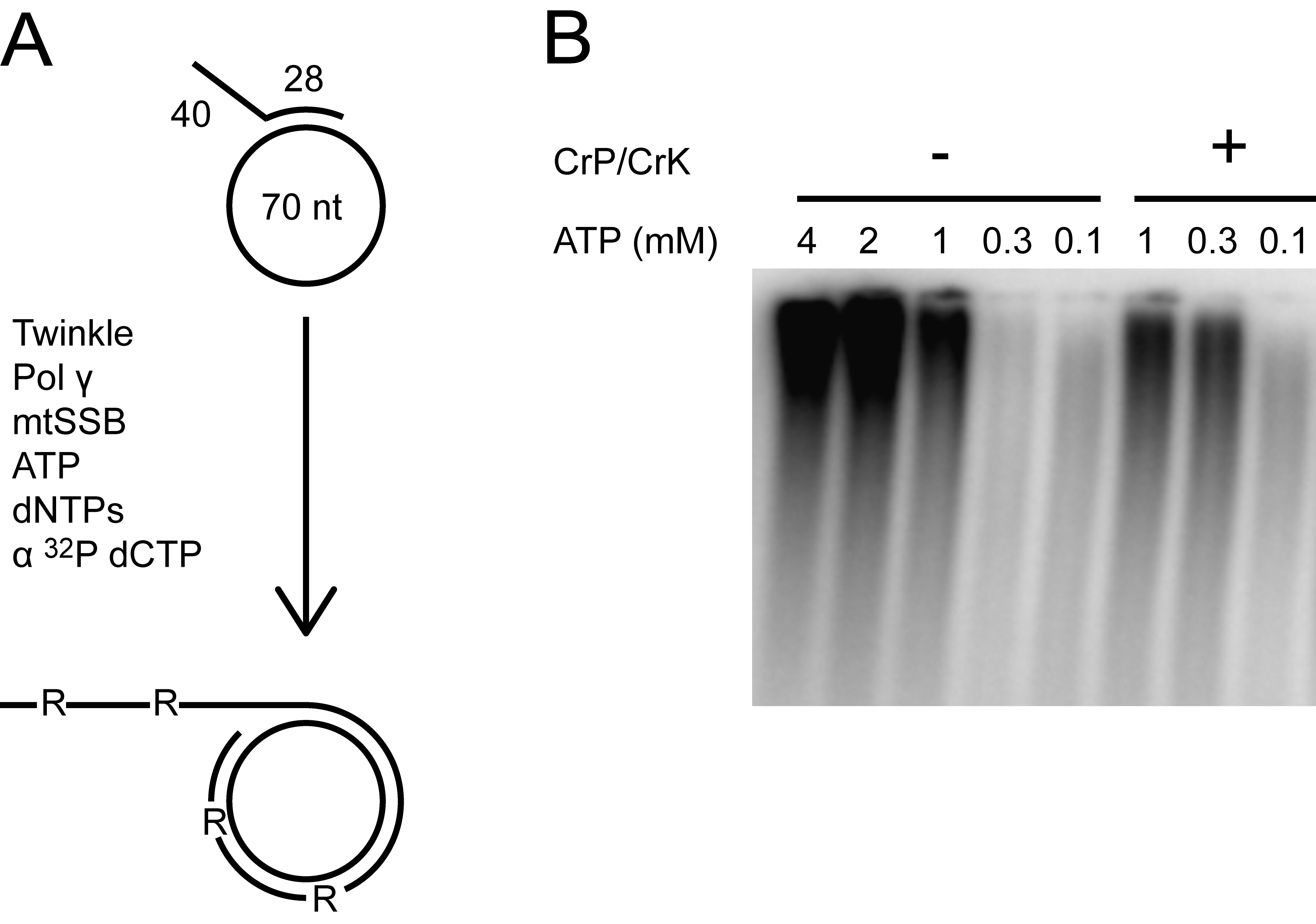

Supplement: S3 Fig — (A) Schematic diagram of the 70 nt ssDNA substrate used to compare the incorporation of rNMPs by Pol γ in the rolling circle replication assay presented in S3B Fig. Replication by the mitochondrial replisome consisting of mtSSB, Twinkle and Pol γ (AB2) on a primed mini-circle substrate with a 5’ overhang for Twinkle loading. (B) Replication in the presence (+) and absence (-) of creatine kinase (400 ng) and 5 mM creatine-phosphate-Tris with the indicated concentration of ATP. Replication products were analysed on a denaturing alkaline agarose gel. (TIF) [file pgen.1007315.s003.tif]

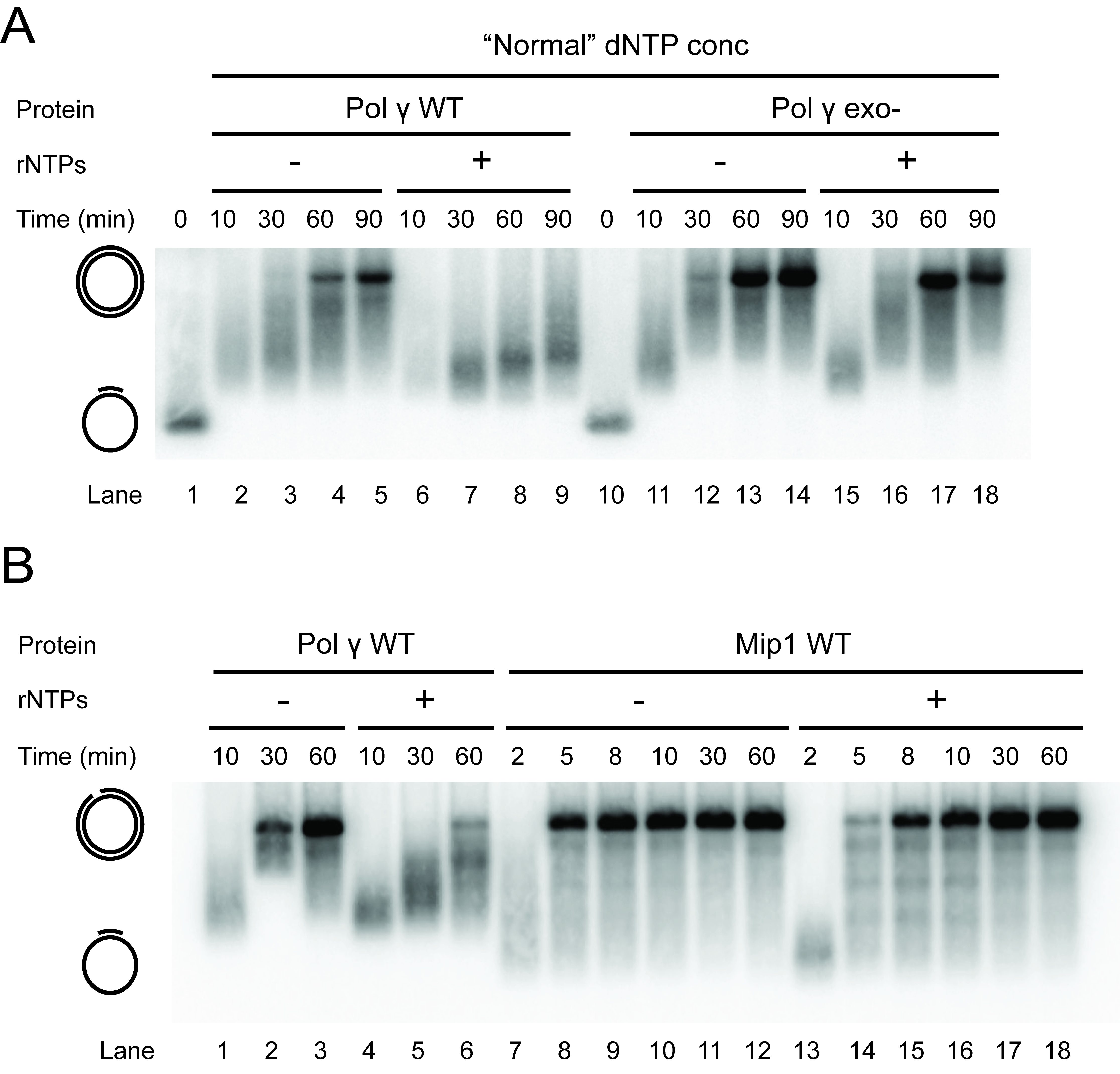

Supplement: S4 Fig — (A) Processivity of WT and exo- Pol γ in “normal” dNTP concentrations in the presence or absence of rNTPs. The 3 kb pBluescript DNA substrate shown in Fig 4A was used and reactions were stopped after the indicated reaction times and separated in an agarose gel electrophoresis. (B) Comparison of processivity by WT Pol γ and yeast mitochondrial DNA polymerase Mip1 on 3 kb pBluescript DNA template. The reactions contained 10 μM dNTP concentrations in the presence or absence of rNTPs. The reactions were stopped at indicated time points and run on an agarose gel. The figure shows a representative picture of two independent experiments. (TIF) [file pgen.1007315.s004.tif]

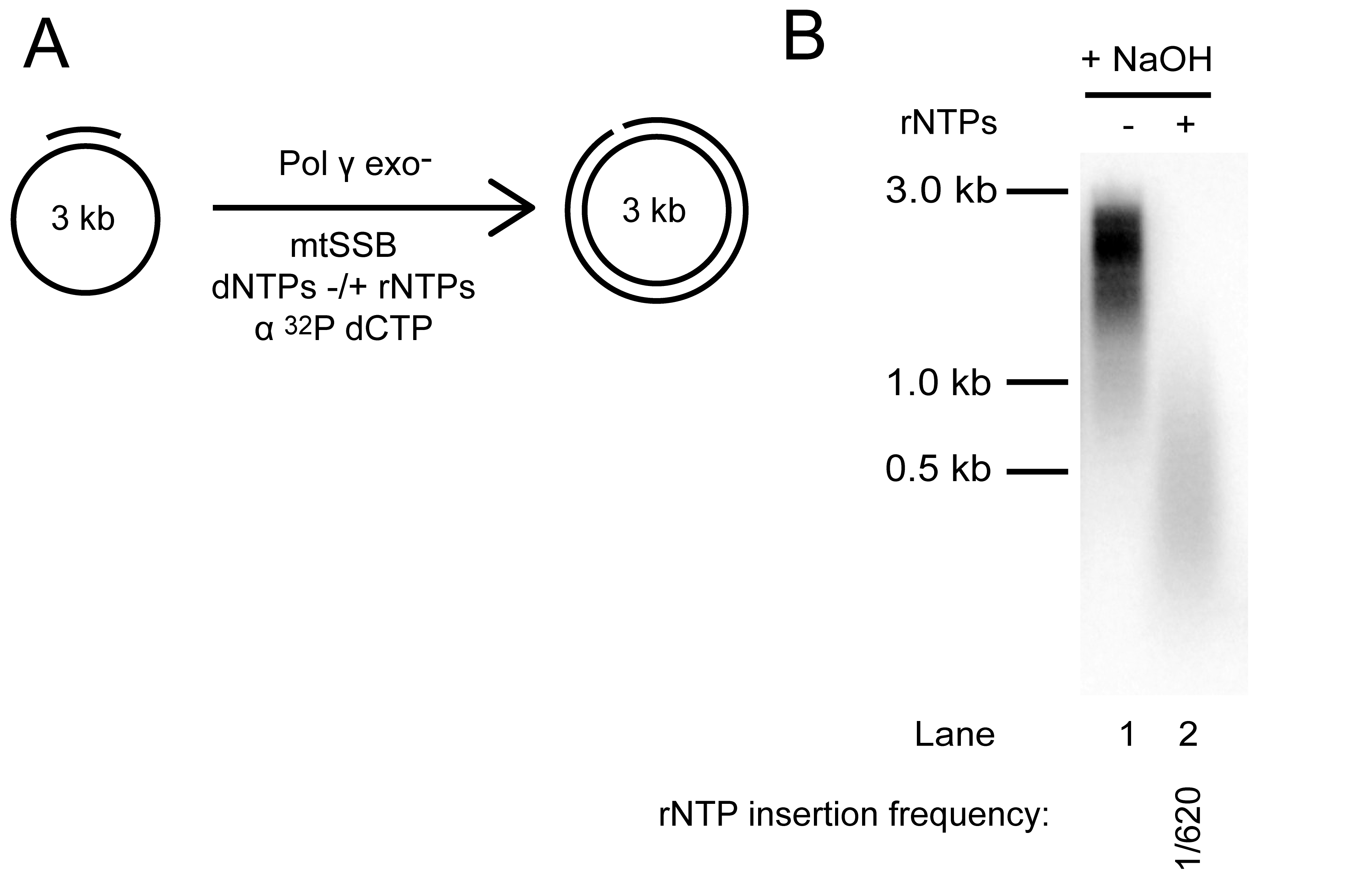

Supplement: S5 Fig — (A) A schematic overview of the incorporation assay shown in (B). (B) Analysis of rNMP incorporation frequency of exo- Pol γ at very low dNTP concentrations (1 μM dATP, 0.5 μM dCTP, 0.5 μM dGTP and 1 μM dTTP) on a 3 kb template. The samples were alkaline treated (+NaOH) and analysed on a denaturing alkaline agarose gel. The rNMP incorporation frequency was determined from the median length of alkali stable products, for more details see Materials and Methods. The incorporation frequency is the average of two independent experiments. (TIF) [file pgen.1007315.s005.tif]
