# Supplementary material for: The presence of rNTPs decreases the speed of mitochondrial DNA replication
Source: PLoS Genet. 2018 Mar 30;14(3):e1007315. doi: 10.1371/journal.pgen.1007315 (PMC5895052; doi:10.1371/journal.pgen.1007315)
Supplement: S1 Table — (PDF) [file pgen.1007315.s006.pdf]

| **Description** | Name and reference | Sequence (5'-3') |
|---|---|---|
| **Primers for double stranded Southern probes** | | |
| Mouse mtDNA 16S rRNA | 1953 Reverse (Tyynismaa et al 2004) | GAG GTG ATG TTT TTG GTA AAC AGG CGG GGT |
| | 1409 Forward | GGT AGA GGT GAA AAG CCT AAC GAG CTT GG |
| | | |
| **Strand specific probes** | | |
| Cytochrome oxidase subunit II H1 | Yao Yang et al 2002, 7591-7618 | TTCTAGGACAATGGGCATAAAGCTATGGTT |
| Cytochrome oxidase subunit II L2 | Yao Yang et al 2002, 7591-7618 | AACCATAGCTTTATGCCCATTGTCCTAGAA |
| D-loop H2 | Yao Yang et al 2002; 15,814-15,840 | CCATCGAGATGTCTTATTTAAGGGGAA |
| D-loop L2 | Yao Yang et al 2002; 15,814-15,840 | TTCCCCTTAAATAAGACATCTCGATGG |
| 16S L3 | nt 1409 Bibb et al 1981 | GGTAGAGGTGAAAAGCCTAACGAGCTTGGT |
| 16S H3 | nt 1409 Bibb et al 1981 | ACCAAGCTCGTTAGGCTTTTCACCTCTACC |
| ND4 L4 | nt 10424 Bibb et al 1981 | TAACGTACTACAAAAACTCTACATCTCAAT |
| ND4 H4 | nt 10424 Bibb et al 1981 | ATTGAGATGTAGAGTTTTTGTAGTACGTTA |
| ND6 L5 | nt 13730 Bibb et al 1981 | ACTTCATCATAATAATTAAGCACACAAATT |
| ND6 H5 | nt 13730 Bibb et al 1981 | AATTTGTGTGCTTAATTATTATGATGAAGT |
| ND1 L6 | nt 3131 Bibb et al 1981 | CTCACTATTCGGAGCTTTACGAGCCGTAGC |
| ND1 H6 | nt 3131 Bibb et al 1981 | GCTACGGCTCGTAAAGCTCCGAATAGTGAG |
| | | |
| **Incorporation primers pBluescript and M13** | | |
| | Primer 682 (Makarova DNA repair 2014) | TATCGATAAGCTTGATATCGAATTCCT |
| | Primer M13 | |
| | | |
| **Oligos for bypass of ribonucleotides** | | |
| | 25 nt primer | ATAGGGGTATGCCTACTTCCAACTC |
| | 70ntNDG | GAGGGGTATGTGATGGGAGGGCTAGGATATGAGGT GAGTT**G**AGTGGAGTTGGAAGTAGGCATACCCCTAT |
| | 70ntNDT | GAGGGGTATGTGATGGGAGGGCTAGGATATGAGGT GAGTT**T**AGTGGAGTTGGAAGTAGGCATACCCCTAT |
| | 70ntNDA | GAGGGGTATGTGATGGGAGGGCTAGGATATGAGGT GAGTT**A**AGTGGAGTTGGAAGTAGGCATACCCCTAT |
| | 70ntNDC | GAGGGGTATGTGATGGGAGGGCTAGGATATGAGGT GAGTT**C**AGTGGAGTTGGAAGTAGGCATACCCCTAT |
| | 70ntrGTP | GAGGGGTATGTGATGGGAGGGCTAGGATATGAGGT GAGTT**(G)**AGTGGAGTTGGAAGTAGGCATACCCCTAT |
| | 70ntrUTP | GAGGGGTATGTGATGGGAGGGCTAGGATATGAGGT GAGTT**(U)**AGTGGAGTTGGAAGTAGGCATACCCCTAT |
| | 70ntrATP | GAGGGGTATGTGATGGGAGGGCTAGGATATGAGGT GAGTT**(A)**AGTGGAGTTGGAAGTAGGCATACCCCTAT |
| | 70ntrCTP | GAGGGGTATGTGATGGGAGGGCTAGGATATGAGGT GAGTT**(C)**AGTGGAGTTGGAAGTAGGCATACCCCTAT |
| | 70nt2rNTPs | GAGGGGTATGTGATGGGAGGGCTAGGATATGAGGT GAGT**(UG)**AGTGGAGTTGGAAGTAGGCATACCCCTAT |
| | 70nt3rNTPs | GAGGGGTATGTGATGGGAGGGCTAGGATATGAGGT GAG**(UUG)**AGTGGAGTTGGAAGTAGGCATACCCCTA T |
| | 70nt4rNTPs | GAGGGGTATGTGATGGGAGGGCTAGGATATGAGGT GA**(GUUG)**AGTGGAGTTGGAAGTAGGCATACCCCTA T |
| | | |
| **Oligos for sequencing of bypass** | | |
| BIOHindIII | Biotinylated primer with internal HindIII site | GTAGAAGCTTGATCTACGAGAGATACTATTAGCTATA GGGGTATGCCTACTTCCAACTC |
| For70ntseq | PCR forward primer | GTAGAAGCTTGATCTACGAGAG |
| Rev70ntseq | PCR reverse primer | GAGGGGTATGTGATGGGAGGGCTAGG |
| M13 rev (-49) | Sequencing primer for pUC19 | GAGCGGATAACAATTTCACACAGG |
| | | |
